# Atracurium Versus Cis-Atracurium for Laryngeal Relaxation and Hemodynamic Stability in Pediatric Patients: A Randomized, Double-Blind Study

**DOI:** 10.7759/cureus.40882

**Published:** 2023-06-24

**Authors:** Arshad Nadirsha, Nandkishore Agrawal, Habib Md R Karim

**Affiliations:** 1 Anaesthesiology, Critical Care, and Pain Medicine, All India Institute of Medical Sciences, Raipur, IND

**Keywords:** safety and efficacy, laryngoscopy and endotracheal intubation, laryngoscopy response, neuromuscular blockers, paediatric anesthesia

## Abstract

Background and aim

Equipotent dose of atracurium and cis-atracurium has failed to show clinically equi-effective muscle relaxation actions required for laryngoscopy and endotracheal intubation (LETI) in adults. There needs to be more data on children. We aimed to compare the efficacy of equipotent atracurium and cis-atracurium for producing optimal LETI. We also compared the hemodynamic stability and side effects.

Methods

With approvals and informed consent, 104 children between three and 12 years were enrolled in the present randomized, double-blind, parallel-arm study. Fifty-two participants were recruited in each group and received either 2ED95 atracurium (0.5mg/kg) or 2ED95 cis-atracurium (0.1mg/kg). Three-point scale, i.e., excellent, good, and poor, were assigned based on jaw relaxation, vocal cords, diaphragmatic movement, coughing, and resistance to the laryngoscope blade. Basic hemodynamics and adverse events like flushing, hemodynamic instability, and airway spasms were noted. The groups were compared using Wilcoxon-Mann-Whitney U or Chi-square tests as applicable; a p-value <0.05 was considered significant.

Results

Entire enrolled participants completed the study. Excellent LETI conditions were significantly higher in the atracurium than in cis-atracurium (53.8% versus 19.2%, p-value <0.001). In the present study, blunted laryngoscopy-related sympathetic surge containing the increase in hemodynamic parameters within 20% from the baseline was noted in both groups, but the blunting and fall back of hemodynamic towards the baseline was rapid in the atracurium (within 7 minutes of LETI) group than cis-atracurium, i.e., 72 minutes; p-value <0.001. Only one flushing was noted in the atracurium group compared to none in the cis-atracurium.

Conclusion

2ED95 dose of cis-atracurium (0.1mg/kg), although have minor advantages of maintaining hemodynamic better, has lower adverse events, it provides significantly lower ‘Excellent LETI conditions’ when compared to 2ED95 dose of atracurium (0.5mg/kg) in children of age three to 12 years.

## Introduction

Muscle relaxation is a crucial component of balanced general anesthesia (GA) which facilitates laryngoscopy and endotracheal intubation (LETI), prevents voluntary and reflex movement, and protects against vocal cord damage [1, 2]. The clinical selection of neuromuscular blocking drugs (NMBDs) is influenced by their speed of onset, duration of action, route of elimination, and associated side effects. Among the NMBDs, nondepolarizing agents are frequently used, especially in the intermediate-acting group. Common skeletal muscle relaxants in pediatric surgery are the newer benzyl isoquinolinium derivatives like atracurium and cis-atracurium [2]. Cis-atracurium is an isomer of atracurium. Although both the drugs are an intermediate-acting and have an onset of action of 3-5 minutes and a duration of action of 20-30 minutes, their ED95 in adults is different, i.e., 0.21 and 0.05 mg/kg, respectively, for atracurium and cis-atracurium. ED95 is a potency measure of NMBDs which is the concentration of the agent which blocks the neuromuscular function of the ulnar nerve by 95% as measured by the muscle twitch height [3]. Cis-atracurium is 3-5 times more potent than atracurium; it is also devoid of histamine-release side effects and has better cardiovascular stability.

However, equipotent doses of atracurium and cis-atracurium have failed to show equi-effectiveness in clinical practice in producing facilitatory LETI conditions. Nevertheless, most previous studies comparing the two drugs involve adults, and the dosage used in pediatric populations is extrapolated from them. There have been a relatively minor number of comparative clinical studies on using cis-atracurium in children [4]. Cis-atracurium infusion was effective in pediatric intensive care units; no adverse effects were noted in the immediate post-infusion period [5]. In the present study, we compared equipotent (2ED95) doses of atracurium and cis-atracurium in pediatric patients undergoing surgeries under balanced GA. The primary objective was to compare the efficacy of atracurium and cis-atracurium for optimal LETI conditions. At the same time, the assessment for the hemodynamic stability, trend, and side effects was done as a secondary objective.

## Materials and methods

Study design and settings

The present prospective, randomized, active-controlled, parallel-group, interventional, double-blind clinical study was conducted in the operating room of an academic institute in India. The study was conducted over two years. The departmental and institutional thesis review committee reviewed the research protocol, followed by approval from the Institute Ethics Committee (AIIMSRPR/IEC/2020/516). Following approval, the study was prospectively registered in the Clinical Trail Registry of India (CTRI/2021/08/035495). The study follows the Declaration of Helsinki and Good Clinical Practice guidelines. Informed and written consent was obtained from the parents or guardians of the patients.

Study population

We enrolled children undergoing surgery under GA with LETI. Inclusion criteria were: children of either male or female gender, aged three to 12 years belonging to the American Society of Anesthesiologists physical status (ASA-PS) class I and II, undergoing elective surgery with an expected duration > 30 minutes. Patients having known allergies to study drugs, airway problems suggestive of difficult intubation, known or suspected neuromuscular diseases, receiving medications that interfere with neuromuscular blockade, and having severe systemic illnesses were excluded. Further, it was also excluded if the patient required more than two laryngoscopy attempts.

After identifying prospective participants and taking written and informed consent, participants were randomly allotted to two groups based on computer-generated random numbers generated from software (http://www.openepi.com). The principal investigator maintained a random number spreadsheet centrally, and patient codes were placed in the case record form; group allocation was concealed by keeping the main spreadsheet in an opaque envelope. Instructions on preparing the drug were also provided to the technician. However, the drug was not disclosed to the person performing the LETI. Both the drugs were diluted to equal volume in a 10ml syringe. A qualified assistant calculated and administered the drug dose per the earlier instructions. The qualified anesthesiologist performing LETI and assessing the effect was blinded to the drug administered as the syringe was only marked as a muscle relaxant.

Sample size

The sample size was estimated using the formula (Z 1-α/2 + Z1-β )2 (p1q1 + p2q2)/2. Data for sample size was taken from a previous study by El-Kasaby et al. [6]. The study showed an excellent response of 37.5% in the atracurium group and 12.5% in the cis-atracurium group; Z 1-α/2=1.96 at a 95% confidence interval, and Z β=0.84 for 80% power. Substituting the values in the formula, the sample size in each group was 44. We also considered a dropout rate of 20% and a design effect of one, which gave a total sample size of 52 patients in each group, a total of 104 with 1:1 allocation.

Technique

All patients underwent pre-anesthesia evaluations before being posted for surgery. An appropriate-size intravenous cannula was secured. All patients were routinely administered antibiotics 30 minutes before surgical incision. Baseline hemodynamic parameters, i.e., systolic blood pressure (SBP), diastolic blood pressure (DBP), mean blood pressure (MBP), and heart rate (HR) were recorded. Entire patients were premedicated intravenously with an injection of midazolam 0.03mg/kg and glycopyrrolate 0.004 mg/kg. Intravenous (IV) GA was induced using an injection of fentanyl 2mcg/kg and an injection of propofol 2mg/kg. A muscle relaxant (study drug) was administered after confirming the ability to mask and ventilate the patient. After 3 minutes, LETI was done proper size tube, and the condition of intubation was assessed and recorded as excellent, good, or poor, based on the degree of relaxation, vocal cord positioning, and intubating responses. GA was maintained with N20, O2, sevoflurane, and intermittent doses of muscle relaxants. The neuromuscular block was reversed by 0.05mg/kg of neostigmine and glycopyrrolate 0.008mg/kg. Patients were clinically monitored for any signs of adverse effects, and if they occurred, they were managed as per standard care and noted in the case record form.

Outcome variables

Hemodynamic parameters and time points of data collection (MBP, HR) recorded at regular timed intervals during surgery T0 before induction (baseline), T1 after induction and before injection of NMBD, T2 after injection of NMBD and before endotracheal intubation T3 just after intubation. Then every 5 minutes for 30 minutes after intubation and every 15 minutes until extubation. Intubation was done by an anesthesia resident having a minimum of two years of experience or a consultant. Intubation condition was assessed using a 3-point scale described in a good clinical practice guideline for pharmacodynamic studies on neuromuscular blockade drugs [7]. The three-point scale was: Excellent - relaxed jaw, abducted immobile vocal cords, and no diaphragmatic movement, no coughing, no resistance to the blade; Good - relaxed jaw, intermediate position, moving vocal cords and slight diaphragmatic movement (bucking), slight resistance to the blade; and Poor - jaw not relaxed, adducted vocal cords, coughing on intubation, active resistance to blade. Patients were clinically monitored for any signs of histamine release through skin changes (graded as flush, erythema, wheal) or hemodynamic changes, bronchospasm, wheeze, increased airway pressure, and desaturation. They were managed as per standard care. HR, blood pressure, SpO2, respiratory rate, temperature, input, and output were monitored and noted.

Data management and statistics

All the raw data were entered into a Microsoft Excel (Microsoft® Corp., Redmond, WA, USA) spreadsheet and analyzed using SPSS version 21 (IBM Corp., Armonk, NY, USA). Numerical data were represented as mean and standard deviation (SD), and categorical data as proportions. The qualitative data were analyzed using the Chi-square test, and quantitative data were analyzed using the Wilcoxon-Mann-Whitney U test, Chi-square test, and other appropriate tests as required. P value ≤ 0.05 was considered statistically significant.

## Results

Entire (N=104, 52 in each group) participants recruited in the study completed the process, and none were excluded from the analysis. Out of 104 patients, 73 were males, and 31 were females. Entire patients were of ASA-PS class I; no comorbidities. The age, gender, weight, and comorbidity were compared. Table 1 shows the number and percentage of patient distribution in different groups.

**Table 1:** Baseline and demographic characteristics of the participants and their comparison. Group A: atracurium, Group C: cis-atracurium. It is to be noted that the statistical significance of peripheral oxyhemoglobin saturation did not have clinical significance.

Parameters	Group A [N=52]	Group C [N=52]	P-value
Age (years)	6.13 (2.56)	5.48 (2.21)	0.210
3-5 Years	25 (48.1%)	29 (55.8%)	0.432
6-12 Years	27 (51.9%)	23 (44.2%)	0.432
Male	33 (63.5%)	40 (76.9%)	0.133
Female	19 (36.5%)	12 (23.1%)	0.133
Weight (kilogram)	19.50 (6.46)	17.70 (5.92)	0.125
Heart rate (per minute)	100.00 (15.81)	101.08 (15.21)	0.724
Systolic blood pressure (mmHg)	102.63 (11.22)	98.96 (9.32)	0.105
Diastolic blood pressure (mmHg)	63.37 (12.10)	59.85 (8.89)	0.125
Mean blood pressure (mmHg)	76.75 (11.22)	73.42 (8.52)	0.134
Respiratory rate (per minute)	24.44 (1.78)	23.88 (2.09)	0.339
Peripheral oxyhemoglobin saturation	99.86 (0.40)	100.00 (0.00)	0.011

The atracurium group had significantly higher excellent LETI conditions than the cis-atracurium group, with a p-value <0.001. The jaw relaxation, vocal cord position and resistance to the tube, coughing/bucking, and limb movements were also favorable for the atracurium group (Table 2).

**Table 2:** Comparison of the laryngoscopy and endotracheal intubation conditions. Group A: atracurium, Group C: cis-atracurium

Parameters	Group A	Group C	Total	χ2	P Value
Condition of Intubation: Excellent	28 (53.8%)	10 (19.2%)	38 (36.5%)	14.166	<0.001
Condition of Intubation: Good	20 (38.5%)	31 (59.6%)	51 (49.0%)		
Condition of Intubation: Poor	4 (7.7%)	11 (21.2%)	15 (14.4%)		
Jaw relaxation: Relaxed	46 (88.5%)	35 (67.3%)	81 (77.9%)	6.755	0.009
Jaw relaxation: Not Relaxed	6 (11.5%)	17 (32.7%)	23 (22.1%)		
Resistance to blade: No Resistance	36 (69.2%)	24 (46.2%)	60 (57.7%)	7.376	0.017
Resistance to blade: Slight Resistance	16 (30.8%)	25 (48.1%)	41 (39.4%)		
Resistance to blade: Active Resistance	0 (0.0%)	3 (5.8%)	3 (2.9%)		
Vocal cord position: Abducted	27 (51.9%)	10 (19.2%)	37 (35.6%)	13.399	0.001
Vocal cord position: Intermediate	21 (40.4%)	30 (57.7%)	51 (49.0%)		
Vocal cord position: Adducted	4 (7.7%)	12 (23.1%)	16 (15.4%)		
Vocal cord movement: Not Moving	29 (58.0%)	15 (31.2%)	44 (44.9%)	7.083	0.008
Vocal cord movement: Moving	21 (42.0%)	33 (68.8%)	54 (55.1%)		
Limb movement: None	36 (69.2%)	17 (32.7%)	53 (51.0%)	13.890	<0.001
Limb movement: Slight	16 (30.8%)	35 (67.3%)	51 (49.0%)		
Coughing/bucking: None	40 (76.9%)	29 (55.8%)	69 (66.3%)	5.211	0.022
Coughing/bucking: Slight	12 (23.1%)	23 (44.2%)	35 (33.7%)		

Both the groups had a fall in HR, SBP, DBP, and MBP following induction until the intubation timepoint, but the differences between the groups were statistically insignificant (Table 3). With laryngoscopy, HR, SBP, DBP, and MBP were increased in both groups. However, the increases were higher in group C (cis-atracurium) compared to group A (atracurium). The changes remained statistically significant from timepoint 5 to 20 minutes for HR and 5 to 30 minutes for MBP (Table 3). The change in HR and MBP over time within each group (Friedman test) was highly significant (p <0.001) for both groups. However, the overall p-value for the comparison of change over time between the two groups (Generalized Estimating Equations) was significant only for MBP, with a p-value of 0.012, but not for HR, with a p-value of 0.375.

**Table 3:** Absolute heart rate and mean blood pressure at different time points and their comparison among the groups. Group A: atracurium. Group C: cis-atracurium.

Timepoints	Heart Rate (beats per minute)			Mean Blood Pressure (mmHg)		
	Group A	Group C	P-value	Group A	Group C	P-value
0 Minutes	100.00 (15.81)	101.08 (15.21)	0.830	76.75 (11.22)	73.42 (8.52)	0.134
1 Minute	100.85 (16.97)	101.25 (15.09)	0.889	73.13 (9.85)	73.52 (7.89)	0.715
2 Minutes	99.83 (17.28)	100.35 (16.29)	0.720	71.67 (9.00)	73.33 (8.93)	0.283
3 Minutes	115.67 (17.72)	120.31 (13.42)	0.068	83.19 (11.39)	86.50 (10.15)	0.074
5 Minutes	110.50 (17.51)	115.17 (12.86)	0.041	78.35 (10.04)	83.48 (9.40)	0.011
10 Minutes	106.96 (15.21)	111.67 (11.40)	0.022	74.71 (10.84)	80.23 (9.55)	0.006
15 Minutes	103.75 (14.91)	108.21 (11.26)	0.023	71.92 (9.87)	77.35 (9.16)	0.006
20 Minutes	101.00 (14.55)	104.60 (10.63)	0.031	71.38 (9.72)	76.27 (8.71)	0.010
30 Minutes	100.54 (14.38)	103.65 (11.79)	0.086	70.77 (10.10)	74.98 (9.21)	0.042
45 Minutes	100.31 (13.82)	100.75 (9.62)	0.588	70.60 (9.36)	73.96 (9.00)	0.162
60 Minutes	100.60 (13.28)	100.79 (8.81)	0.519	69.65 (13.87)	74.33 (8.38)	0.057
75 Minutes	100.57 (9.58)	101.96 (9.43)	0.364	71.26 (9.33)	75.20 (7.64)	0.112
90 Minutes	100.34 (10.36)	102.98 (9.61)	0.156	73.07 (9.99)	76.45 (8.74)	0.147
105 Minutes	101.45 (11.19)	102.79 (9.61)	0.442	75.13 (12.49)	79.82 (7.98)	0.242
120 Minutes	104.06 (14.02)	104.33 (9.73)	0.934	76.53 (9.90)	79.22 (7.89)	0.618
135 Minutes	101.78 (16.74)	103.11 (9.85)	0.535	72.89 (10.52)	79.33 (8.47)	0.121
150 Minutes	100.38 (14.46)	101.00 (5.18)	0.398	71.12 (16.15)	81.17 (4.83)	0.261

The p-value for change over time within each group (Friedman test) for SBP for both groups was <0.001. The same for the DBP was 0.004 for group A and <0.001 for group C. Nevertheless, the comparison of the change in SBP over time between the two groups (Generalized Estimating Equations) was not significant for both SBP (p-value 0.076) and DBP (p-value 0.181). The maximum change from the zero minutes time point was observed at the three minutes (just after LETI) for all, i.e., HR, SBP, DBP, and MBP. The comparisons for SBP and DBP are presented in Table 4.

**Table 4:** Absolute systolic and diastolic blood pressure values across different time points and their comparison among the groups Group A: atracurium. Group C: cis-atracurium.

Timepoints	Systolic Blood Pressure (mmHg)			Diastolic Blood Pressure (mmHg)		
	Group A	Group C	P-value	Group A	Group C	P-value
0 Minutes	102.63 (11.22)	98.96 (9.32)	0.105	63.37 (12.10)	59.85 (8.89)	0.125
1 Minute	98.25 (9.88)	99.12 (9.37)	0.527	58.75 (10.93)	59.98 (8.90)	0.597
2 Minutes	97.19 (8.40)	99.12 (10.37)	0.410	57.29 (9.84)	59.79 (9.59)	0.260
3 Minutes	110.90 (12.29)	115.04 (11.52)	0.066	68.90 (12.24)	73.29 (10.92)	0.038
5 Minutes	104.54 (11.95)	110.17 (10.41)	0.018	63.40 (11.51)	69.75 (10.76)	0.003
10 Minutes	100.71 (11.35)	106.63 (9.54)	0.010	60.02 (12.47)	66.81 (10.00)	0.003
15 Minutes	97.38 (9.78)	103.13 (9.11)	0.005	57.69 (11.66)	63.90 (10.79)	0.003
20 Minutes	96.31 (10.11)	99.79 (15.54)	0.020	57.33 (11.63)	62.98 (10.08)	0.006
30 Minutes	96.15 (10.09)	100.02 (10.40)	0.036	57.00 (11.42)	62.52 (9.90)	0.014
45 Minutes	95.98 (8.95)	99.25 (9.26)	0.134	57.08 (10.42)	61.73 (10.44)	0.054
60 Minutes	95.75 (10.06)	98.98 (8.62)	0.106	57.90 (11.38)	62.06 (8.75)	0.027
75 Minutes	96.57 (9.16)	100.22 (8.40)	0.102	57.72 (10.86)	62.62 (8.42)	0.055
90 Minutes	98.22 (9.65)	102.14 (8.80)	0.093	59.66 (11.21)	63.86 (9.25)	0.107
105 Minutes	101.70 (12.55)	105.14 (8.58)	0.419	61.40 (12.83)	66.93 (8.45)	0.117
120 Minutes	103.24 (10.00)	104.61 (8.18)	0.973	63.29 (9.32)	66.33 (8.06)	0.444
135 Minutes	98.67 (11.95)	104.89 (8.13)	0.225	60.44 (10.71)	66.89 (9.12)	0.148
150 Minutes	97.00 (16.12)	108.00 (7.59)	0.149	58.88 (15.92)	67.83 (5.67)	0.346

**Figure 1:**
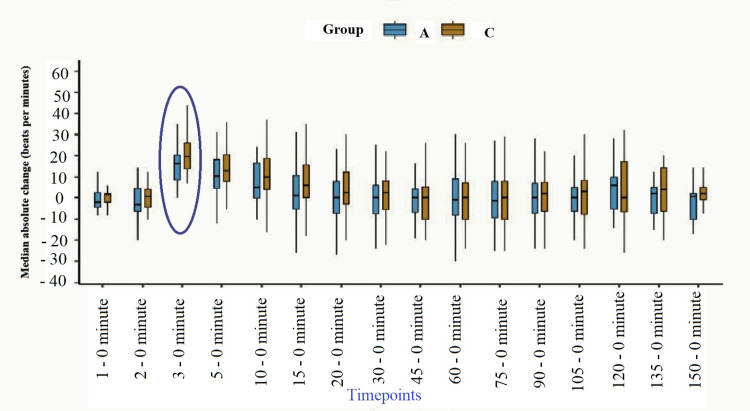
Median absolute change in heart rate from baseline at different time points among the groups and their comparison. Encircled time point differences were statistically significant.

The mean (SD) absolute change in the heat rate within group A from the baseline was significantly higher for three-to-zero and five-to-zero minutes time points. In contrast, for group C, it was significant for three-to-zero, five-to-zero, and ten-to-zero minute differences. However, comparing the mean (SD), absolute change among groups A and B showed a significant difference only for three-to-zero minutes timepoints. The median absolute changes from the baseline among groups A and C are shown in Figure 1.

On comparison of the mean (SD), percent changes from baseline within the group, the SBP, DBP, and MBP were significantly greater for three-to-baseline (zero minutes) timepoints for group A (atracurium), but the same was significant for 3, 5, and 10 minutes from the baseline for group C (cis-atracurium). Nevertheless, the comparison of the mean (SD) percent changes between the group showed that group C (cis-atracurium) had persistently increased SBP, DBP, and MBP from one to 75 minutes (Table 5).

**Table 5:** Mean (SD) percentage change in the Systolic, Diastolic, and Mean blood pressure from the baseline within and among the groups and their comparison. SD: standard deviation, Group A: atracurium, Group C: cis-atracurium.

Timepoint Comparison	Systolic Blood Pressure (mmHg)					Diastolic Blood Pressure (mmHg)					Mean Blood Pressure (mmHg)				
	Group: A		Group: C		P-value (Group A versus C) for the difference from zero minutes to follow-up time points	Group: A		Group: C		P-value (Group A versus C) for the difference from zero minutes to follow-up time points	Group A		Group C		P-value (Group A versus C) for the difference from zero minutes to follow-up time points
	Mean (SD) of % Change	P-value of Change Within Group	Mean (SD) of % Change	P-value of Change Within Group		Mean (SD) of % Change	P-value of Change Within Group	Mean (SD) of % Change	P-value of Change Within Group		Mean (SD) of % Change	P-value of Change Within Group	Mean (SD) of % Change	P-value of Change Within Group	
1 - 0 Minutes	-3.6% (10.3)	0.956	0.3% (4.7)	1.000	0.003	-4.62 (10.96)	0.998	0.13 (3.78)	1.000	0.014	-3.8% (11.4)	0.994	0.5% (7.0)	1.000	0.011
2 - 0 Minutes	-4.6% (9.3)	0.587	0.3% (6.3)	1.000	<0.001	-6.08 (12.03)	0.844	-0.06 (5.81)	1.000	<0.001	-5.5% (12.7)	0.891	0.1% (8.2)	1.000	0.004
3 - 0 Minutes	9.0% (14.1)	<0.001	16.7% (11.2)	<0.001	0.002	5.54 (14.81)	<0.001	13.44 (9.36)	<0.001	0.001	9.9% (17.1)	<0.001	18.5% (13.1)	<0.001	0.008
5 - 0 Minutes	2.9% (14.9)	0.356	11.8% (10.5)	<0.001	0.001	0.04 (15.90)	0.202	9.90 (8.81)	<0.001	<0.001	3.9% (17.8)	0.217	14.3% (11.8)	<0.001	0.002
10 - 0 Minutes	-0.8% (14.5)	1.000	8.3% (10.5)	0.002	<0.001	-3.35 (17.47)	1.000	6.96 (9.34)	<0.001	<0.001	-0.9% (17.8)	1.000	9.8% (11.4)	<0.001	0.001
15 - 0 Minutes	-4.2% (12.6)	0.899	4.8% (10.5)	0.764	<0.001	-5.67 (16.41)	1.000	4.06 (8.83)	0.276	<0.001	-4.8% (15.6)	0.998	5.9% (11.4)	0.248	<0.001
20 - 0 Minutes	-5.3% (11.9)	0.625	1.2% (16.0)	1.000	<0.001	-6.04 (15.45)	1.000	3.13 (7.76)	0.535	<0.001	-5.7% (14.6)	0.997	4.4% (10.5)	0.625	<0.001
30 - 0 Minutes	-5.5% (11.9)	0.483	1.5% (10.5)	1.000	0.004	-6.37 (15.07)	0.986	2.67 (8.24)	0.973	0.002	-6.4% (15.3)	0.903	2.7% (12.0)	0.998	0.003
45 - 0 Minutes	-5.7% (11.1)	0.505	0.7% (8.9)	1.000	0.002	-6.29 (14.74)	0.996	1.88 (8.84)	0.990	0.003	-6.6% (15.1)	0.920	1.3% (11.3)	1.000	0.009
60 - 0 Minutes	-6.0% (11.2)	0.411	0.4% (7.6)	1.000	0.003	-5.46 (13.63)	0.998	2.21 (7.93)	0.998	0.005	-8.3% (18.6)	0.697	1.8% (10.2)	1.000	0.002
75 - 0 Minutes	-3.9% (10.5)	0.998	0.9% (7.9)	0.996	0.031	-3.78 (13.25)	1.000	2.76 (8.03)	0.454	0.030	-4.0% (13.6)	1.000	2.6% (10.4)	0.859	0.021
90 - 0 Minutes	-2.4% (12.6)	1.000	2.8% (10.7)	0.535	0.064	-2.02 (12.96)	0.999	3.90 (8.47)	0.015	0.027	-1.9% (14.8)	1.000	4.0% (11.7)	0.081	0.072
105 - 0 Minutes	0.8% (14.7)	0.237	4.4% (11.1)	<0.001	0.262	-0.23 (15.41)	0.016	5.64 (8.81)	<0.001	0.224	1.1% (18.1)	0.042	7.4% (12.4)	<0.001	0.199
120 - 0 Minutes	3.0% (15.0)	<0.001	6.7% (11.1)	<0.001	0.498	-0.88 (15.95)	<0.001	4.67 (8.27)	<0.001	0.477	1.0% (18.4)	<0.001	7.5% (11.7)	<0.001	0.541
135 - 0 Minutes	0.1% (17.4)	<0.001	3.3% (9.5)	<0.001	0.965	-1.22 (16.41)	<0.001	6.00 (4.77)	<0.001	0.476	-1.6% (18.7)	<0.001	4.9% (6.9)	<0.001	0.860
150 - 0 Minutes	-2.2% (20.3)	<0.001	5.5% (8.7)	<0.001	0.650	-4.00 (21.59)	<0.001	7.17 (8.82)	<0.001	0.362	-5.2% (24.6)	<0.001	7.6% (9.8)	<0.001	0.362

The mean (SD) of the duration of surgery, intraoperative fluid administration and blood loss, and the need for the supplemental dose of muscle relaxants were statistically indifferent among the groups (Table 6).

**Table 6:** Comparison of surgery duration, fluid administered, and blood loss among the groups. Group A: atracurium, Group C: cis-atracurium

Parameters	Wilcoxon-Mann-Whitney U Test			
	Group- A	Group- C	W	p-value
Duration of surgery (minutes)	106.15 (31.83)	104.42 (26.40)	1353	0.997
Total Intra-operative fluids (mL)	144.42 (52.88)	144.33 (53.46)	1378	0.868
Total Intra-operative blood loss (mL)	28.23 (11.62)	25.60 (9.97)	1497	0.342
Number of doses of relaxant administered	4.13 (1.96)	3.69 (1.78)	1515	0.284

## Discussion

The present study compared the equipotent doses (2ED95) of atracurium and cis-atracurium and found that atracurium provided better relaxation, laryngoscopy, and tracheal intubation conditions. Our finding is like El-Kasaby et al.’s study [6]. The authors also compared 2ED95 doses of both atracurium and cis-atracurium in adult patients undergoing abdominal surgeries. They found that atracurium had a more effective neuromuscular blocking agent and provided good intubation conditions than cis-atracurium. They also compared 4ED95 and 6ED95 cis-atracurium with 2ED95 atracurium and noted that only higher doses of cis-atracurium, like 4ED95 and 6ED95, provided excellent intubation conditions comparable to 2ED95 atracurium. Littlejohn et al. observed well to the excellent intubating condition following 0.5mg/kg atracurium in 95% of the patients [8].

In contrast, an equipotent dose of cis-atracurium 0.1mg/kg provided only acceptable intubating conditions in two-thirds of the patients [8]. We also noted similar findings in our pediatric cohort. It suggests that equipotent in terms of ED95 of atracurium and cis-atracurium did not translate into clinically equi-effective results, and a higher time of ED95 of cis-atracurium might be required to produce a similar intubating condition compared to 2ED95 of atracurium in pediatrics. Narang et al., however, noted a different result in adult patients undergoing abdominal laparoscopic surgeries and found that the equipotent dose of cis-atracurium provided a better laryngoscopy condition [9]. It might be because the authors have used 0.3mg/kg of atracurium, which is lower than the 2ED95 dose, and compared with 0.1mg/kg cis-atracurium, which is the 2ED95 dose. For laryngoscopy and intubation, at least a 2ED95 dose is required and usually practiced [10]. When 0.5mg/kg atracurium and 0.1mg/kg cis-atracurium were compared by Paśko-Majewska et al. for the same type of surgery, the authors noted that cis-atracurium could not provide excellent laryngoscopy and intubation condition for most of the patients [11]; our results corroborate with it. On the other hand, when 0.15mg/kg (3ED95) of cis-atracurium was compared to the 0.5mg/kg atracurium, it was noted that the cis-atracurium was able to provide optimal and comparable intubating conditions [12]. The discrepancy between the clinical finding in the table when equipotent dosages were used, that also for a drug and its isomer, reiterates that ED95 might not be the best potency measure for neuromuscular drugs as suggested by Kopman et al. [13].

Cis-atracurium is an isomer of atracurium, and both are non-depolarizing neuromuscular blocking drugs. A larger dose of atracurium releases histamine causing hypotension and tachycardia, thus leading to cardiovascular instability. Although cis-atracurium has nearly similar onset and action duration, it lacks histamine release and has better cardiovascular stability [14]. In the present study, blunted laryngoscopy-related sympathetic surge containing the increase in hemodynamic parameters within 20% from the baseline was noted in both groups. Still, the blunting was better in the atracurium group than in cis-atracurium.

Further, the positive shift of the HR and BPs for the atracurium got settled within seven minutes of intubation. However, the positive shift in the cis-atracurium group remained significant until 75 minutes (72 minutes from LETI). It might be due to the atracurium group's underlying histamine-induced vasodilation and blood pressure fall. Histamine release side effects were graded as flush, erythema, wheals, wheeze, bronchospasm, increased airway pressures, and desaturation. One of the patients in the atracurium group had flushed at the injection site, but no such side effects were noted in the cis-atracurium group. Lien et al. also noted no adverse cardiovascular effects and no histamine release up to eight times ED95 (0.4mg/kg) of cis-atracurium [14]. Littlejohn et al. also do not show histamine release with cis-atracurium [8]. Flushing was noted in two out of 20 patients given atracurium (0.5mg/kg) by Bluestein et al. [15]. Lepage et al. interpreted that up to 5ED95 of cis-atracurium, there was no change in the plasma histamine concentration, and no skin manifestations were noted [16]. Cis-atracurium has also shown its efficacy in maintaining hemodynamics in patients with low left ventricular function undergoing cardiac surgeries [17].

The study has some limitations. It was done in a single tertiary care center, so the possibility of hospital bias could not be ruled out. Intubating conditions have been assessed only clinically at all time points. Using neuromuscular monitoring in pediatric patients with different morphological characteristics was not always possible but could have improved the assessment of adequate intubating conditions. Although a single senior anesthesiologist performed the intubation in all patients, there may be variations between patients. Lastly, we considered 2ED95 doses of drugs for adults but used them in the pediatric population. Recent studies have shown that the ED95 is age dependent [18, 19], and it might impact the interpretation and conclusion we reached.

## Conclusions

2ED95 of atracurium (0.5mg/kg) provided a more effective, excellent intubation condition than an equipotent dose of cis-atracurium (2ED95, i.e., 0.1mg/kg). However, hemodynamic stability was comparable in both groups. Histamine-related side effects were noted in one patient with atracurium and none with cis-atracurium. Hence, a 2ED95 dose of cis-atracurium fails to provide an excellent intubation condition, and a higher dose of cis-atracurium may be required.
